# Plastid Phylogenomics of *Camphora officinarum* Nees: Unraveling Genetic Diversity and Geographic Differentiation in East Asian Subtropical Forests

**DOI:** 10.3390/ijms26189229

**Published:** 2025-09-21

**Authors:** Chen Hou, Yingchao Jiang, Qian Zhang, Jun Yao, Huiming Lian, Minghuai Wang, Peiwu Xie, Yiqun Chen, Yanling Cai

**Affiliations:** Guangdong Provincial Key Laboratory of Silviculture, Protection and Utilization, Guangdong Academy of Forestry, Guangzhou 510520, China; houchen@sinogaf.cn (C.H.); 18218632491@163.com (Y.J.); zhangq7610@sinogaf.cn (Q.Z.); yaojun@sinogaf.cn (J.Y.); lhming@sinogaf.cn (H.L.); wangmh@sinogaf.cn (M.W.); xiepeiwu@sinogaf.cn (P.X.); chenyiqun8012@163.com (Y.C.)

**Keywords:** camphor tree, chloroplast genome, phylogeny, nucleotide polymorphism, simple repeat sequences

## Abstract

*Camphora officinarum* Nees constitutes a pivotal tree species within the evergreen broad-leaved forests of East Asia, endowed with significant economic, ornamental, and ecological importance. Nevertheless, previous research has markedly underestimated the genetic diversity of this species, thereby hindering our efforts in conserving resources and enhancing genetic breeding. The current study generated 155 chloroplast genomes from specimens of *C. officinarum* obtained from six provinces/regions in China. The results reveal the identification of seven distinct clades (I–VII), with Clades II, III, V, and VII exhibiting genome expansions, primarily influenced by lineage-specific elongation of inverted repeats (IRs), whereas Clades I, IV, and VI maintained conserved IR lengths. Despite the structural plasticity, the GC content remained highly conserved. Geographic patterns indicated gene flow between adjacent regions (e.g., Hunan and Hubei with identical IR lengths), but genetic isolation in Fujian. High-polymorphism regions (*psba*-*matK*, *ycf1*, *ycf2*, and *ndhF*) were identified as superior phylogenetic markers, enhancing intraspecies-level resolution. Simple sequence repeats (SSRs) varied significantly among clades, dominated by A/T-rich mononucleotide repeats. These repeats, along with divergent repeat types (e.g., absence of reverse repeats in Clades V/VI), serve as robust tools for resource identification and evolutionary trajectory inference. Phylogenetically, samples from Fujian formed a distinct lineage, while samples from other regions, especially Guangdong, were mixed, with this finding probably being a reflection of historical cultivation and anthropogenic translocation. This study offers a framework for the genetic breeding and investigation of the evolutionary history of *C. officinarum*.

## 1. Introduction

Chloroplast DNA (cpDNA) in plants harbors abundant genetic variation due to its crucial and conserved roles, playing a pivotal role in the study of evolutionary relationships [1,2]. Relative to plant nuclear and mitochondrial genomes, chloroplast genomes exhibit a high degree of conservation in terms of structure, gene content, and sequence organization [3,4]. Compared to protein coding genes, the non-coding region of cpDNA contains more evolutionary variations and lacks functional significance, rendering it particularly valuable for the study of genetic architecture and evolutionary history [2,4,5]. For example, Lu et al. [6] utilized chloroplast genome analysis to investigate evolutionary relationships among three *Cardiocrinum* (Liliaceae) species. Zhai et al. [7] conducted phylogenetic analysis on chloroplast genome samples from 35 species across 31 Ranunculaceae genera, clarifying taxonomic issues for 11 controversial tribes within the family. Their research also demonstrated two evolutionary events in Ranunculaceae’s development, with most extant tribes and genera originating from these events. Zhang et al. (2018) employed chloroplast genome data to construct a phylogenetic tree for Hubei crabapple (*Malus hupenhensis*) using the maximum likelihood method [8]. With respect to these studies, an in-depth analysis of the chloroplast DNA data facilitates a more comprehensive understanding of the evolutionary history of the eudicots family [9,10,11].

The phylogeny of Lauraceae, a family within the magnoliids of eudicots, has captured our attention in recent years. Upon comparing the plastomes of nine species in the Lauraceae family, it was found that differential contractions of the canonical inverted repeat (IR) led to the presence of two distinct types of IRs within the family [12]. A phylogenetic analysis of 24 genera of Lauraceae emphasizes the monophyletic nature of Lauraceae, supported by nine well-supported branches using cpDNA data [13]. The identification and disclosure of six plastome types revealed that at least two separate instances of loss occurred at the boundary between the inverted repeat (IR) and the Large Single-Copy (LSC) regions, and an independent expansion of the Small Single-Copy (SSC) region, during the evolution of the Lauraceae family’s plastomes [14]. The occurrence of substantial plastome enlargement within the tribe Cinnamomeae was reported using cpDNA data. The results indicate that these plastomes of Cinnamomeae have an adequate number of parsimony-informative sites appropriate for molecular dating purposes [15]. The original genus *Cinnamomum* of Lauraceae was classified into two genera, *Cinnamomum* and *Camphora* Fabr., by utilizing cpDNA, nrITS, and plastid *psbA*-*trnH* sequences [16]. Phylogenetic analysis of cpDNA in a more recent study, however, showed that *Camphora* was not monophyletic, while the ribosomal DNA dataset supported the monophyly of *Camphora* [17]. The phylogenetic position of a newly described species *Cinnamomum guizhouense* (C. Y. Deng, Zhi Yang & Y. Yang) was recently reported using chloroplast genome analysis [18]. Thus, chloroplast genomes serve as fundamental resources for the examination of plant genetic diversity, which is essential for species conservation, resource exploration, and sustainable development of Lauraceae plants.

*Camphora officinarum* Nees, a member of the Lauraceae family and an evergreen broadleaf tree species, plays a pivotal role in the community structure of East Asian subtropical evergreen broadleaf forests [19,20,21,22,23]. The timber derived from this species is utilized for the manufacture of furniture and paper, underscoring its substantial ecological significance and economic utility [19,24]. The leaves of *C. officinarum* are rich in volatile terpenoids, which are extensively utilized in medicinal applications, the fragrance industry, and various industrial processes [25,26,27,28,29,30]. Nevertheless, the populations of this species have experienced a marked decline in the wild, primarily due to the impacts of climate change and anthropogenic disturbances [31]. This situation underscores the critical importance of conducting genetic analyses to assess the germplasm diversity of *C. officinarum*, which is essential for its scientific conservation and sustainable exploitation. Earlier studies classified the Chinese *C. officinarum* into two main groups [19,20] or three groups [21] by means of nuclear EST-SSRs, genome resequencing, and genotyping by sequencing on natural populations. However, these findings, while partially elucidating the evolutionary history, conflict with the broad geographical distribution observed across multiple provinces. This discrepancy highlights the inadequacy of such simplified genetic classifications from a breeding perspective, obfuscating the intricate genetic relationships inherent in camphor evolution. Additionally, the absence of a systematic approach to identify efficient molecular markers for inter- and intra-specific identification of Lauraceae species further complicates future hybridization and breeding efforts.

This study compared the assembly and annotation of 155 chloroplast genomes from six provinces in South China (Figure S1A). A phylogenetic tree was constructed using the maximum likelihood method to identify divergent groups of *C. officinarum* in South China. Nucleotide polymorphisms were analyzed to locate regions of high variability within the plastid genomes of each group. This study also investigated repetitive elements, their distribution, and clade-specific features across different groups of *C. officinarum*. These results offer a theoretical foundation for the development of molecular markers and hybrid breeding techniques for *C. officinarum*.

## 2. Results

### 2.1. Fundamental Attributes of the Chloroplast Genomes in Camphor Trees

**Figure 1:**
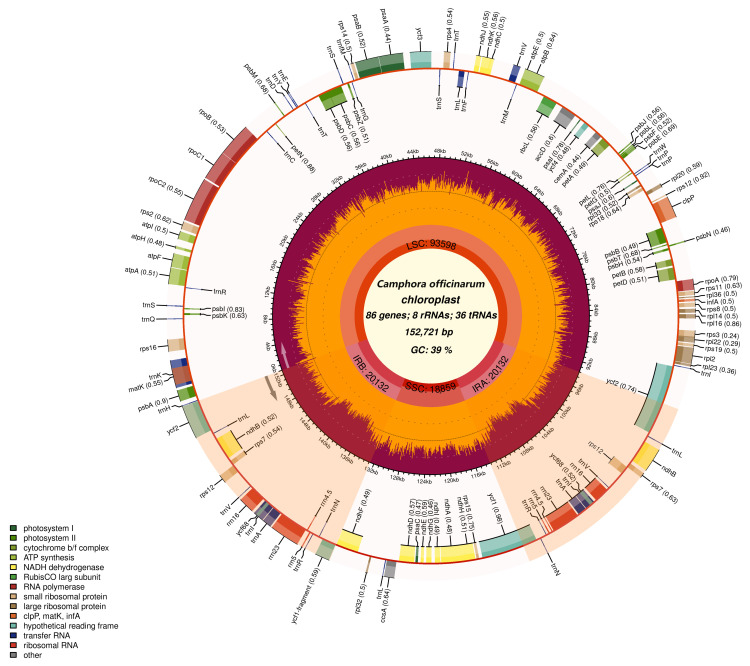
Circular map of the chloroplast genome of *C. officinarum*. The innermost ring displays core genomic metrics: total length (152,721 bp), GC content (39%), and functional gene counts (86 genes, including 8 rRNAs and 36 tRNAs). The second ring annotates major structural regions: Large Single-Copy (LSC, 93,598 bp, orange), Small Single-Copy (SSC, 18,899 bp, purple), and Inverted Repeat A (IRa, 20,132 bp, red). The outer rings depict gene locations color-coded by functional categories: photosynthesis (green), ribosomal proteins (large: yellow; small: light yellow), ATP synthase (blue), NADH dehydrogenase (red), cytochrome c oxidase (orange), RNA polymerase (pink), tRNAs (black), rRNAs (brown), and other genes (ycf, matK, infA; gray). Gene orientations are indicated by transcriptional direction (clockwise/counterclockwise).

Upon the assembly of chloroplast genomes from 155 camphor trees sourced from various geographical locations, it was ascertained that these genomes invariably encompass 130 genes. These genes comprise 84 protein-coding genes (CDS), 36 transfer RNA (tRNA) genes, and 8 ribosomal RNA (rRNA) genes. Categorization by function reveals that the 130 genes consist of 44 genes associated with the photosynthetic system, 60 genes involved in transcriptional and translational processes, 5 genes related to biosynthesis, and 4 genes with functions yet to be elucidated (Figure 1 and Table 1).

### 2.2. Phylogenetic Analysis Utilizing the Maximum Likelihood Method

**Figure 2:**
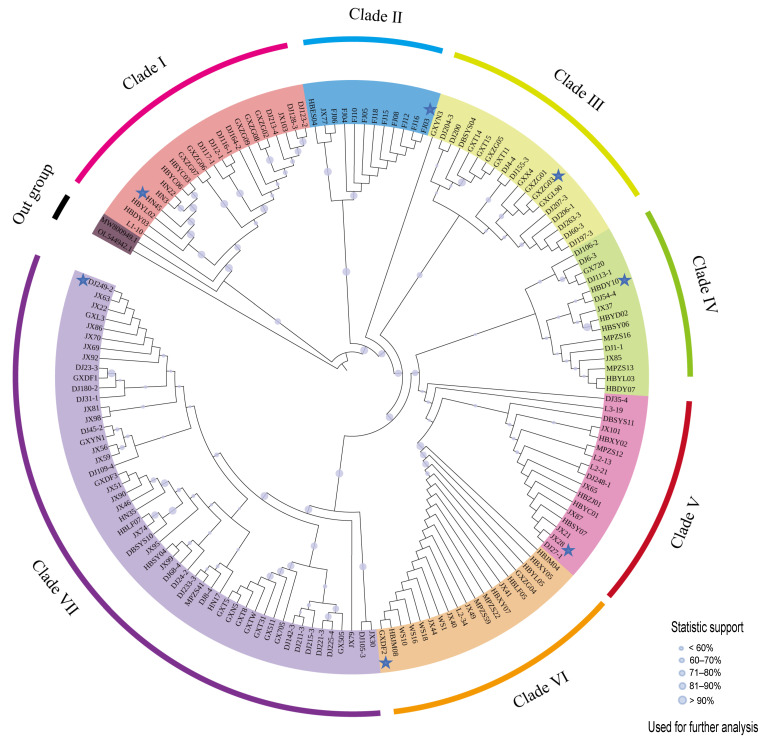
Phylogenetic reconstruction of *C. officinarum* chloroplast genomes. The maximum likelihood tree, derived from complete chloroplast genome sequences, illustrates the evolutionary interrelationships among seven principal clades (I–VII), each denoted by a distinct color. The tree is annotated with statistical support values, which are represented by circles of varying diameters. Samples designated for subsequent analysis are marked with blue stars.

The phylogenetic trees were constructed using a dataset consisting of 155 chloroplast genome sequences of *C. officinarum*. The 155 specimens were distributed across seven clades, designated as Clades I through VII (Figure 2). Within these clades, Clade VII had the highest number of specimens, with 52, followed by Clade I (21), Clade III (19), Clade VI (19), Clade V (17), Clade IV (15), and the smallest clade, Clade II (12). Notably, Clade II was predominantly (representing 83% of this clade) composed of specimens from Fujian Province. In contrast, specimens from other provinces did not exceed 50% within each clade (Figure S1B). The intermingling of specimens from six provincial regions across these clades prevented the determination of geographical distribution patterns for the camphor tree species. Consequently, we selected one representative chloroplast genome from the innermost portion of each clade (HN45, HBDY10, GXZG03, DJ249-2, JX28, GXDF2, and FJ03) to represent the genetic diversity of *C. officinarum* originating from seven representative specimens for subsequent analytical procedures.

### 2.3. Comparative Analysis of Chloroplast Genome Boundaries in Camphor Trees

The chloroplast genomes of seven representative specimens of *C. officinarum* exhibit a typical quadripartite structure, indicating a high degree of conservation in terms of total sequence length and the lengths of distinct regions (Table 2). The total sequence length varies between 152,620 and 154,078 base pairs, with the LSC region spanning 93,565 to 93,688 base pairs, the SSC region ranging from 18,859 to 18,894 base pairs, and the IR region measuring between 40,148 and 41,620 base pairs. The GC content also displays considerable conservation (Table 2): the total GC content ranges from 39.1% to 39.2%, the LSC region from 37.9% to 38.0%, the SSC region from 33.8% to 33.9%, and the IR region from 44.3% to 44.4%. Despite the relatively conserved sequence lengths, GC content, genomic structure, and gene lengths observed in the chloroplast genomes of camphor leaves, significant differences in the boundaries of the LSC, SSC, and IR regions are evident across the seven distinct phylogenetic lineages.

**Figure 3:**
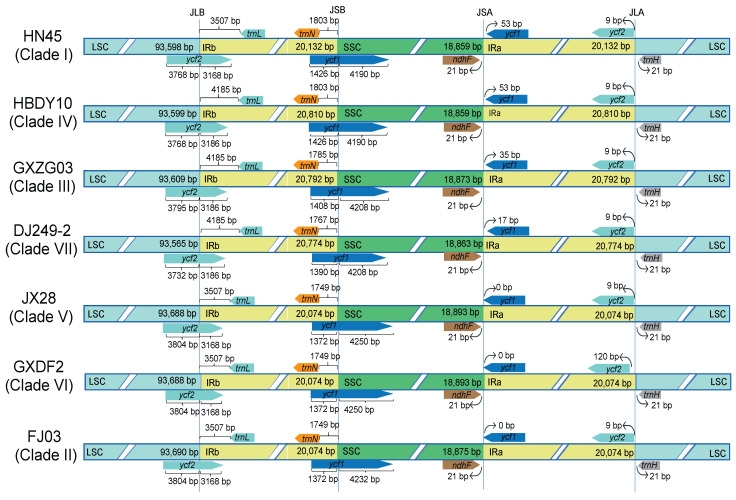
Comparative analysis of section boundaries of plastid genomes across seven representative specimens of *C. officinarum*. Linearized maps depict structural variations at junctions between the Large Single-Copy (LSC), Small Single-Copy (SSC), and inverted repeat regions (IRa/IRb).

Research has revealed that at the demarcation between the LSC and IRb regions, the chloroplast genomes of JX28, GXDF2, and FJ03 exhibit an insertion of 3804 base pairs (bp) within the LSC region, whereas GXZG03 displays an insertion of 3795 bp (Figure 3). HN45 and HBDY10 manifest an insertion of 3768 bp, while DJ249-2 contains an insertion of 3732 bp. Within the IRb region, the *ycf2* gene on the branches of HBDY10, GXZG03, and DJ249-2 measures 3186 bp, in contrast to 3168 bp for the branches of HN45, JX28, GXDF2, and FJ03. At the IRb-SSC boundary, the length of *ycf1* varies considerably: HN45 and HBDY10 exhibit 1426 bp, GXZG03 1408 bp, DJ249-2 1390 bp, and JX28, GXDF2, FJ03 1372 bp. Within the SSC region, the length of *ycf1* ranges from 4190 bp for HN45 and HBDY10, to 4208 bp for GXZG03 and DJ249-2, 4250 bp for JX28 and GXDF2, and 4232 bp for FJ03. The expansion/contraction patterns observed at the SSC/IRa and IRa/LSC junction regions are similar. The *ycf1* genes from the seven representative specimens are all situated in the IRa region, each spanning 1372 bp. Specifically, HN45 and HBDY10 are 53 bp from the SSC/IRa boundary; GXZG03’s *ycf1* gene is 35 bp from this boundary; DJ249-2’s *ycf1* gene is 17 bp away, while JX28, GXDF2 and FJ03’s *ycf1* genes are all contiguous with the boundary. Additionally, the *ndhF* genes from the seven representative specimens are all located in the SSC region, and the *trnH* genes are in the LSC region. Notably, both *ndhF* and *trnH* genes are 21 bp away from their respective IRa regions. Although *trnL* and *trnN* genes are all in the IRb region across all seven species, their distances from the LSC and SSC regions differ. For example, the *trnL* genes in HN45, JX28, GXDF2, and FJ03 are 3507 bp from the LSC/IRb boundary, whereas those in HBDY10, GXZG03, and DJ249-2 are 4185 bp away. The *trnN* genes of HN45 and HBDY10 are located 1803 bp from the IRb/SSC boundary, while GXZG03’s *trnN* gene is 1785 bp away. DJ249-2’s *trnN* gene shows a distance of 1767 bp, whereas JX28, FJ03, and GXDF2’s *trnN* genes are 1749 bp from the IRb/SSC boundary. These findings indicate that the *trnL* and *trnN* genes in the IRb region demonstrate significant variations in their distances from the IRb/LSC and IRb/SSC boundaries across the seven representative specimens, while the genes (*ndhF* and *trnH*) maintain relatively stable positions. The differentiation in *ycf2* and *ycf1* gene lengths, along with the drift patterns of *trnL* and *trnN* genes, suggest potential expansion/contraction phenomena in the IR region. Variations in boundary distances between LSC, SSC, IRa, and IRb may influence genomic stability and recombination efficiency.

### 2.4. Sequence Similarity Analysis of the Seven Representative Specimens

**Figure 4:**
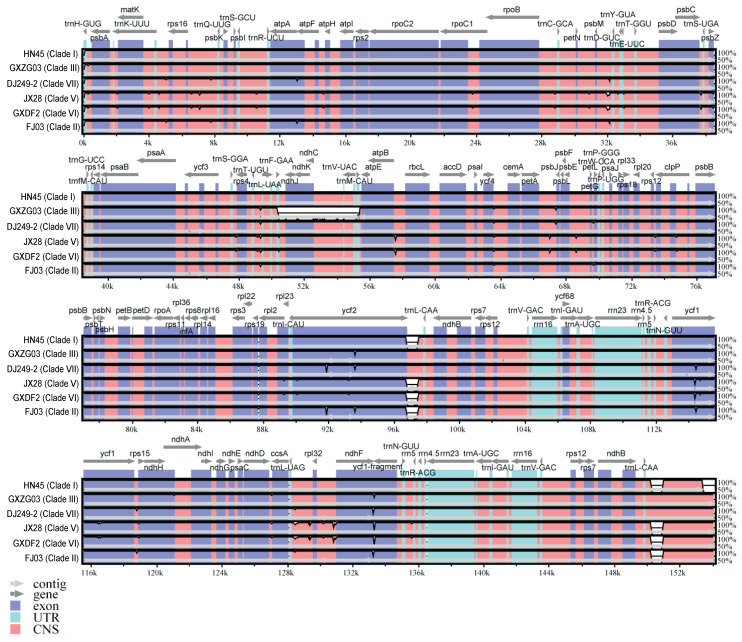
Comparative chloroplast genomes of seven representative specimens of *C. officinarum* using mVISTA. The mVISTA plot illustrates sequence conservation of chloroplast genomes across the seven representative specimens of *C. officinarum*, aligned against a reference sequence HBDY10. The X-axis indicates genome position (0–152,721 bp), annotated with gene names (e.g., *matK*, *ycf1*). The *Y*-axis indicates percent identity (50–100%) of sequence similarity. Different color code different regions: exons (blue-purple), UTRs (untranslated region, cyan), CNS (conserved non-coding sequences, pink), and non-coding regions (gray).

The chloroplast genomes of these species predominantly exhibit variations in gene intercalation regions, with minor differences in gene sequences (Figure 4). Notable discrepancies are evident in genes such as *ccsA*, *ndhA*, *ndhD*, *rdhG*, *rps16*, *ycf1*, and *ycf2*. GXDF2 and JX28 exhibit more pronounced differences in comparison to other chloroplast genome sequences, particularly at positions 6–74 k, 75–95 k, and 112–137 k. Notably, significant sequence discrepancies are present at positions 6–7.5 k, 31–34 k, 56.5 k, 66.5–68 k, 91.5–93 k, and 128–131 k. HN45 displays a relatively high degree of similarity to HBDY10. The chloroplast genome sequences of GXZG03, DJ249-2, and FJ03 exhibit a higher degree of similarity, while the seven representative specimens primarily differ in sequence fragments at 12–13.5 k, 31–32.5 k, and 91.5–93 k. The variations are mainly distributed in the LSC and SSC regions, with significant differences primarily occurring in the non-coding regions of gene intervals, while the coding regions remain relatively conserved. Notably, the most pronounced variations are observed in the 91.5–93 kb (*ycf2*) and 113.5–115 kb (*ycf1*) regions within the coding areas.

### 2.5. Analysis of Nucleotide Polymorphism in the Camphor Chloroplast Genome

**Figure 5:**
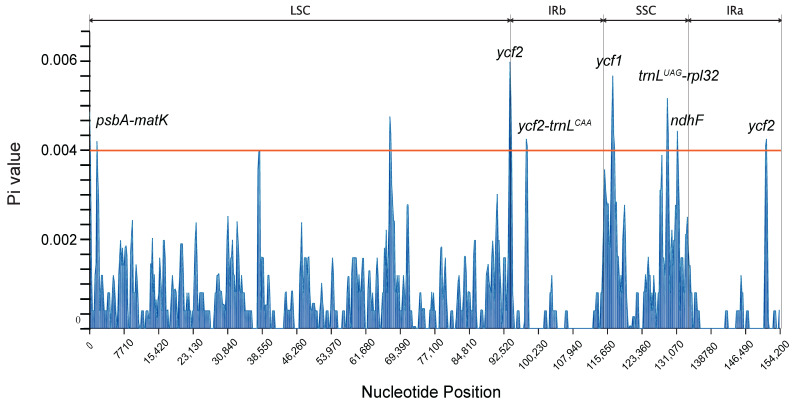
Statistical map for the analysis of nucleotide polymorphisms in the chloroplast genome of *C. officinarum*. Sliding window analysis was applied to calculate Pi values across the entire chloroplast genome. Structural regions are labeled: LSC (Large Single-Copy), IRb (Inverted Repeat B), SSC (Small Single-Copy), and IRa (Inverted Repeat A). Key gene loci are annotated.

Utilizing DNAsp software, a nucleotide polymorphism (Pi) analysis was executed on 156 sequences of the camphor leaf chloroplast genome, accompanied by the visualization and statistical analysis of the outcomes (Figure 5). The Pi values were observed to fluctuate between 0 and 0.00594, with a maximum value of 0.00563 at the *ycf2* gene situated at the LSC-IRb boundary. The investigation pinpointed regions exhibiting high variability, characterized by Pi values surpassing 0.004, encompassing the following: *psbA*-*matK*, *ycf2*, *ycf2*-*trnL^CAA^*, *ycf1*, *trnL^UAG^*-*rpl32*, *ndhF*, and *ycf2*. Specifically, these regions and their respective Pi values are as follows: *psbA*-*matK* within the LSC region (Pi 0.00417); *ycf2* at the LSC-IRb boundary (Pi value 0.00594); *ycf2*-*trnL^CAA^* within the IRb region (Pi value 0.00422); *ycf1* between the SSC region (Pi 0.00563); *trnL^UAG^*-*rpl32* and *ndhF* within the SSC region (Pi values 0.00513 and 0.00401, respectively); and *ycf2* within the IRa region (Pi value 0.00422).

### 2.6. Comparative Analysis of Chloroplast Repetitive Sequences of Camphor Tree

**Figure 6:**
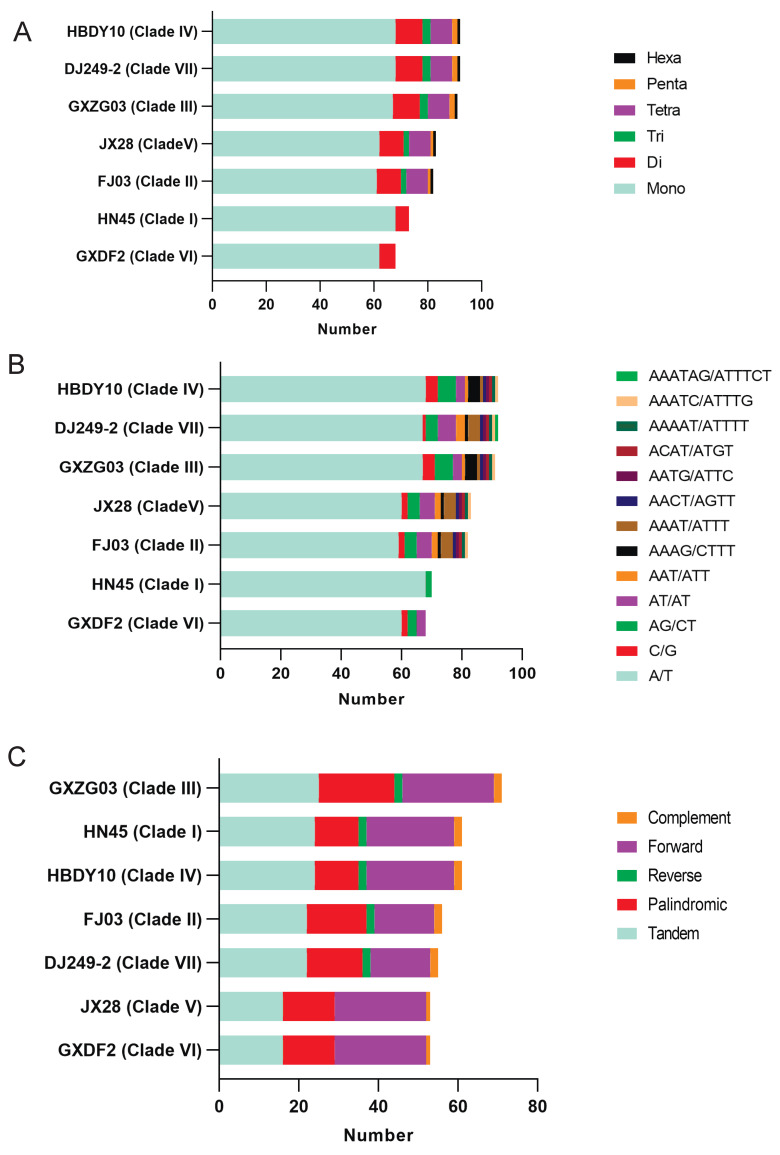
Comparative analysis of simple sequence repeats (SSRs) and repetitive sequence types among the seven representative specimens of *C. officinarum*. (**A**) Numbers of simple sequence repeats (SSRs); (**B**) number of duplication series motif types considering sequence complementary; (**C**) number of repetitive sequence types. Abbreviations: Mono (mononucleotide), Di (dinucleotide), Tri (trinucleotide), Tetra (tetranucleotide), Penta (pentanucleotide), and Hexa (hexanucleotide).

The results indicate that all seven representative specimens of *C. officinarum* contain six types of nucleotide repeats. Specifically, DJ249-2 and HBDY10 exhibit the highest number of simple sequence repeats (SSRs), with a total of 92. Conversely, GXDF2 displays the fewest SSRs, with 68 occurrences (Figure 6A). Among the six identified types of SSRs, mononucleotide repeats are the most common, accounting for 78.4% of all SSRs, with a typical range of 61 to 68 SSRs across the seven specimens. Dinucleotide repeats account for 10.2% of the SSR types, ranging from 5 to 10 repeats among the seven specimens. Trinucleotide repeats are present in three copies in DJ249-2, HBDY10, and GXZG03; in two copies in JX28 and FJ03; and are absent in HN45 and GXDF2. Tetranucleotide repeats are present in the five specimens (DJ249-2, HBDY10, GXZG03, JX28, and FJ03), with eight occurrences each, whereas none are found in GXDF2 and HN45. Pentanucleotide repeats occur twice in DJ249-2, HBDY10, and GXZG03 and once in JX28 and FJ03, with none present in HN45 and GXDF2. Hexanucleotide repeats are found in DJ249-2, HBDY10, GXZG03, JX28, and FJ03, but not in HN45 or GXDF2.

The study identified a total of 13 types of duplication series motifs, taking into account sequence complementarity (Figure 6B). DJ249-2 and HBDY10 have the highest number of duplication series motifs, with a total of 92 instances. Conversely, GXDF2 displays the fewest SSRs, with just 68 instances. Specifically, in terms of sequence motifs, the A/T motif type predominates at 77.7% of the total, followed by AG/CT (5.0%), AT/AT (4.3%), C/G (2.6%), and AAAT/ATTT (2.6%). It is noteworthy that the A/T type has a typical range of 59 to 68 pieces among the seven specimens, with the highest abundance in HBDY10 and HN45 and the lowest in FJ03. Specimens HBDY10 and GXZG03 displayed the highest abundance of the C/G type, with four pieces each. This type occurred twice in JX28, FJ03, and GXDF2, once in DJ249-2, and was absent in HN45. The chloroplast genomes of all seven representatives contained two types of dinucleotide repeat sequences, namely AG/CT and AT/AT, which possess typical ranges of two to six and zero to five, respectively. Five specimens (HBDY10, DJ249-2, GXZG03, JX28, and FJ03) shared a common trinucleotide repeat sequence—the AAT/ATT motif—with a typical range of one to nine repeats. In contrast, none were found in HN45 and GXDF2. Five distinct patterns of tetranucleotide repeats were identified—AAAG/CTTT, AAAT/ATTT, AACT/AGTT, AATG/ATTC, and ACAT/ATGT—with AAAT/ATTT being the most prevalent. Pentanucleotide repeats were limited to two types, AAATC/ATTTG and AAAAT/ATTTT, each found in five representative specimens (HBDY10, DJ249-2, GXZG03, JX28, and FJ03), whereas none were found in HN45 and GXDF2. The hexanucleotide repeat sequence AAATAG/ATTTCT was exclusively present in the chloroplast genomes of DJ249-2, whereas no such sequence was detected in the other specimens.

The plastid genomes of seven representative specimens of *C. officinarum* exhibit considerable variation in the quantity of repetitive sequence types (Figure 6C). Repetitive sequences are categorized into five types: tandem, palindromic, direct, inverted, and complementary repeats. Among the five types, tandem repeats constitute the largest proportion at 36.4%, followed by forward repeats at 34.8%, palindromic repeats at 23.4%, complementary repeats at 2.9%, and reverse repeats at 2.5%. In terms of the total number of repetitions, three distinct levels are evident. GXZG03 (71 pieces), HBDY10 (61 pieces), and HN45 (61 pieces) exhibit the highest number of repetitions; FJ03 follows with 56, and DJ249-2 has 55; GXDF2 and JX28 have the fewest, each with 53. Regarding tandem repeats, GXZG03 has the highest number, with 25 occurrences, whereas JX28 and GXDF2 have the fewest, with 16 each. The number of palindromic repeats ranges from 11 to 19, and the number of forward repeats ranges from 15 to 23 among the seven specimens. It is noticeable that GXDF2 and JX28 lack reverse repeats, whereas the other five specimens possess them. Compared to GXDF2 and JX28, each with one specimen, the other five specimens—GXZG03, HN45, HBDY10, FJ03, and DJ249-2—exhibit a greater abundance of complementary repeats, with two specimens each, in their chloroplast genomes.

## 3. Discussion

Camphor trees, which are widely distributed throughout China, are indigenous evergreen broadleaf species native to the southern provinces. The extensive dispersal of *C. officinarum* potentially enriches genetic variations across diverse geographical regions. In the present study, 155 camphor tree samples from six southern Chinese provinces/regions were assembled and annotated. A phylogenetic tree was constructed to facilitate the comparison of structural features, sequence discrepancies, and chloroplast repeat sequences across the seven representative specimens of *C. officinarum*. The present study aims to explore the potential genetic differentiation within the Chinese camphor tree populations.

The chloroplast genomes of seven clades (I–VII) exhibit notable structural variations compared to previously published references. While total genome lengths in prior studies (e.g., Chen et al., 2017 [32]: 152,570 bp; Li et al., 2019 [33]: 152,729 bp) largely align with Clades I, IV, and VI (152,729 bp), Clades II, III, V, and VII display significant expansions (152,620–154,078 bp), primarily driven by inverted repeat (IR) lengthening (e.g., Clade V JX28: 41,620 bp vs. Qiu et al., 2020 [34]: 39,936 bp). This IR expansion—up to 1688 bp longer than the reference—suggests lineage-specific genomic rearrangements in *C. officinarum*. Despite structural plasticity, GC content remains highly conserved across all samples (total GC: 39.1–39.2%; IR GC: 44.2–44.4%), mirroring reference values (Wu et al., 2017 [12]: 39.1%; Li et al., 2019 [33]: 39.2%). Such stability implies strong functional constraints on nucleotide composition, particularly in IR regions where GC-rich rRNA operons reside. Geographically, samples from Hunan (HN45, Clade I) and Hubei (HBDY10, Clade IV) share identical genomic metrics (152,729 bp total, 40,148 bp IR). In contrast, Fujian accessions diverge in IR structure (40,192 bp vs. 40,148 bp). These findings underscore dynamic chloroplast evolution, where IR length polymorphism—rather than point mutations—drives clade differentiation within *C. officinarum*. Notably, Clade II (FJ03) uniquely combines reduced total length (152,620 bp) with elevated IR length (40,192 bp), indicating compensatory SSC/LSC contractions. In angiosperm chloroplast genomes, the contraction and expansion of boundaries between IR regions and LSC or SSC regions are widespread phenomena, as documented in previous studies [35,36,37]. The rates of synonymous substitution within the IR region of the genes are significantly reduced, generally exhibiting a rate that is 3.7 times slower than those observed in LSC and SSC regions of the plastid genomes of *C. officinarum*. This likely contributes to the contraction and expansion of the IR regions across the seven representative specimens of *C. officinarum*. Our result is congruent with the prevalent occurrence of contraction and expansion of the IR regions within the Lauraceae family [13,14,15].

Sequence variations in chloroplast genomes have long been utilized for plant taxonomy and evolutionary studies due to lack of recombination and moderate nucleotide evolution rates. They serve as examples of resolution of the monophyly of Cinnamomum (*Cinnamomum* spp.) based on *trnL*-*trnF* chloroplast DNA sequences [38], the phylogeny of Lauraceae was also resolved using several plastid segments [39]. In the present study, several high-polymorphism regions in *C. officinarum—ycf2*, *ycf1*, and *ndhF*—are reported, and they may serve as phylogenetic markers for further genetic breeding. The results of our similarity analysis revealed that sequence variations are predominantly distributed in intergenic regions (e.g., 6–7.5 k, 91.5–93 k) and coding regions (e.g., *matK*, *rps16*, *ccsA*), with minimal variation in coding regions. Notably, the SSC regions of *C. officinarum* chloroplast genomes are predominantly high-polymorphism areas (Pi > 0.2), containing genes such as *matK*, *ycf1*, *ccsA*, and *ndh*, significantly enhancing inter- and intraspecies resolution capabilities in future phylogenetic studies. Moreover, transcriptomic analysis showed that *matK* gene expression increased significantly under both high- and low-temperature stress in cucumber, further proving that cucumber chloroplasts respond to temperature stress by regulating lipid and ribosome metabolism. The *accD* gene has higher chances of DNA editing under high-temperature stress, which may improve heat tolerance in cucumber [40]. Further research is recommended to focus on the expression of the *MatK* and *ccsA* genes in plastid genomes of *C. officinarum* to ascertain whether genetic variations are temperature-dependent across various regions of in South China.

The presence of simple sequence repeats and repetitive sequences underscores their efficacy for phylogenetic studies and genetic breeding of *C. officinarum*. Our findings indicate that DJ249-2 harbors the highest number of SSRs (96), while JX28/GXDF2 possesses the least (83); the latter is congruent with the number (83 SSRs) reported in *Cinnamomum camphora* (=*C. officinarum*) [32]. Examination of dispersed and tandem repeats has demonstrated that GXDF2 and JX28 possess fewer copies compared to FJ03, DJ249-2, and GXZG03, and notably, GXDF2 and JX28 are devoid of reverse repeats. These variations may indicate distinct evolutionary trajectories across the seven representative specimens of *C. officinarum*. In the current study, mononucleotide repeats constitute 60% to 68.5% of the total number of repeat elements, with A/T predominating at 98.3% and C/G at 1.7% of the total mononucleotide repeats. This outcome is largely consistent with the previous observation that mononucleotide repeats constitute nearly 60% of the total SSRs, with A/T motifs comprising 74.5%, which corresponds with the chloroplast’s AT bias [32]. The utilization of these repetitive sequences establishes a basis for additional research into the genetic breeding of camphor trees.

This study identified seven major phylogenetic branches: 155 camphor tree species sources were divided into seven lineages, with significant genetic differentiation observed in Fujian, Jiangxi, and Guangxi (e.g., FJ03, JX28, GXDF2), while populations in other provinces showed genetic mixing likely influenced by historical migration or hybridization. Our findings of the current study do not align with those in previous studies, which categorized the Chinese *C. officinarum* into two primary groups [19,20] or three distinct categories [21]. Camphor trees in other areas especially in Guangdong, display less differentiation, potentially due to China’s extensive history of cultivation and wide-ranging artificial introduction and planting [31]. Camphor trees settled in East Asian subtropical evergreen broadleaf forests during the early Miocene, with accelerated diversification rates in later stages, likely linked to habitat heterogeneity caused by intensified East Asian monsoons. Compared to endemic camphor trees in China, post-glacial isolation drove genetic divergence (Japan vs. China/Taiwan) and diversity loss in Japan, while anthropogenic transfer of plant materials caused large gene flow between cultivated and native local populations. Detected immigrations also led to localized hybridization, altering regional gene pools [31]. The exploration of sequence variation in chloroplast genome enables us to uncover the population genetics and evolutionary history of *C. officinarum*.

## 4. Materials and Methods

### 4.1. Material Collection

Between the years 2022 and 2023, a comprehensive two-year field study was executed by the research team across six distinct regions: Fujian Province, Guangdong Province, Guangxi Zhuang Autonomous Region, Hunan Province, Hubei Province, and Jiangxi Province. The investigation was centered on the distribution of camphor tree resources within these areas, during which clean and healthy leaf samples (~2 g) were procured from 155 individuals. All data amassed, encompassing the geographical origins of the samples, are documented in Table S1. All samples were dried and subsequently preserved over a long period of time within an ultra-low temperature freezer set at −80 °C.

### 4.2. Genome DNA Sequencing and Assembly Annotation

Extraction of high-quality genomic DNA from the 155 samples was performed using the Hi-Pure Plant Genomic DNA Kit (Tsingke Biotechnology Co., Beijing, China), adhering to the guidelines of the manual. To guarantee the quality and purity of extracted DNA, optical density (OD) values were measured. The Qubit 3.0 Fluorometer dsDNA HS Assay Kit (Invitrogen, Carlsbad, CA, USA) was utilized to measure the DNA concentration, and the distribution of DNA fragment size was evaluated by 1% agarose gel electrophoresis. Using HiSeq 3000 sequencing platform (Illumina Inc., San Diego, CA, USA), paired-end reads of 150 bp were sequenced through genome skimming.

After sequencing, all raw data were subjected to the removal of low-quality reads and adaptors using Trimmomatic v0.39 [41]. The clean data were further used for assembling chloroplast genomes using GetOrganelle software v.1.6.0 [42]. A previously published camphor tree plastid genome (NC_035882.1) was downloaded via the NCBI database (http://blast.ncbi.nlm.nih.gov/) (accessed on 16 March 2023) and used as the reference genome. With the reference genome, Software Bandage v0.81 [43] and Gepard v1.40 [44] were used to examine circular structures and right directions for assembly of plastid genomes. After plastid genome assembly, a thorough annotation process was performed using GeSeq (https://chlorobox.mpimp-golm.mpg.de/geseq.html, accessed on 21 August 2025) [45]. Subsequent manual verification and refinement were conducted using Geneious Prime v2022.2.2 [46]. To correct potential annotation errors—including gene omissions, duplicated sequences, aberrant intron structures, misaligned start/stop codons, and inaccurate gene/exon boundaries—thereby guaranteeing the precision and integrity of the genomic annotation.

### 4.3. Construction of the Phylogenetic Tree

Before constructing a phylogenetic tree, sequence alignment based on the 155 samples was performed using MAFFT v7.467 [47] (see Supplementary S1 in the Supplementary Materials). Two plastid genomes, representing *Cinnamomum japonicum* Siebold (accession number OL544942.1) and *Cinnamomum wilsonii* Gamble (accession number MW800949.1), were downloaded from the NCBI database as the outgroup. The phylogenetic tree of *C. officinarum* was constructed using the maximum likelihood (ML) method implemented in the IQ-TREE software v2.1.1 [48]. The GTRGAMMA model and 1000 bootstrap replicates were set to evaluate the statistical support of each phylogenetic branch. The obtained phylogenetic tree with statistical support was further visualized using iTOL v6.4 [49].

### 4.4. Chloroplast Nucleotide Polymorphism Analysis

Nucleotide polymorphisms, quantified as nucleotide diversity (denoted by pi values), were analyzed across both protein-coding and non-coding segments of the 155 genomes using DnaSP v5 [50]. The parameters were set to include a sliding distance of 200 base pairs per step and a window length of 600 base pairs. The outcomes were depicted in the form of line graphs. Additionally, sequence similarity was visualized using mVISTA v2.12 [51], with HBDY10 designated as the reference sequence. The Shuffle-LAGAN mode was implemented to enhance the identification of structural rearrangements, and a window size of 100 base pairs was established to achieve a balance between sensitivity to local variations and computational efficiency. The identity threshold was set at 50% to ensure the identification of divergent regions.

### 4.5. Chloroplast Genome Structure and IR/SC Boundary Analysis

The circular map of the chloroplast genome of *C. officinarum* was generated using OGDRAW software v1.3.1 [52]. Genome size, lengths of the LSC, SSC, and IRs were measured using Geneious Prime software. The GC content of the entire plastid genome, as well as within each region, was measured using Editseq software v1.6.0 installed in Lasergene v5.0.1 [53]. The IR/LSC and IR/SSC boundaries were visualized using CPJSdraw v1.158 [54].

### 4.6. Simple Repeat Sequence Analysis

The examination of simple repeat sequences within the chloroplast genome of *C. officinarum* was performed using MISA-web v1.4.0 [55]. The parameters were set as follows: occurrences for mononucleotide repeats were established at 10; for dinucleotide repeats, 5; for trinucleotide repeats, 4; and for tetranucleotide, pentanucleotide, and hexanucleotide repeats, occurrences were uniformly set to 3. After obtaining the relevant data, we used Excel software to create bar charts illustrating the frequencies of simple repeat sequences, providing a more intuitive visual representation for the analysis of the data.

Additionally, the REPuter software v3.0 [56] was utilized to conduct a thorough identification of repetitive sequences within the chloroplast genome. This included positive repeats, complementary repeats, reverse repeats, and palindromic repeats. The parameters were set as follows: the length of repetitive base units was set at 30 base pairs (bp), the Hamming distance was set to 3, the minimum repeat length was maintained at 30 bp, and the maximum number of displayed repeats was limited to 1000. All other parameters were set to the software’s default values.

To conduct an in-depth analysis of tandem repeats within the sequence, we utilized the online identification tool, Tandem Repeat Finder v2.2.119 [57]. During the analysis, we chose the basic mode and executed the identification process with the tool’s default parameters to ensure accuracy and uniformity.

## 5. Conclusions

The construction of a phylogenetic tree using chloroplast genomes from 155 camphor specimens of *C. officinarum* revealed seven main lineages (Clades I–VII). Clades II, III, V, and VII had genome elongation due to inverted repeat region (IR) expansion, while Clades I, IV, and VI showed genomic stability. Despite the presence of IR expansion and contraction across the seven representative specimens, the GC content is highly conserved globally (39.1–39.2%) and in IR regions (44.2–44.4%), indicating strong functional selection pressure. In chloroplast genomes, regions like *ycf1*, *ycf2*, and *ndhF* are high-variability zones, with polymorphism levels 3–5 times greater than low-variability genes like *rbcL*. Sequence variations are mainly in non-coding regions and specific coding genes, with the SSC region (Pi > 0.2) serving as a polygenic hotspot. Specimens from Fujian form a separate lineage (Clade II), probably due to habitat isolation, while populations in Guangdong and other regions have less genetic divergence, possibly due to historical cultivation and anthropogenic translocation. This study explains the structural and sequence variation of plastid genomes of *C. officinarum*, providing a theoretical basis for designing molecular markers and hybrid breeding in the future.

## Figures and Tables

**Table 1:** Basic information of *C. officinarum* chloroplast genome.

Category	Gene Functions	Genes
Photosynthesis	Photosynthem I	*psaA*, *psaB*, *psaC*, *psaI*, *psaJ*
	Photosynthem II	*psbA*, *psbB*, *psbC*, *psbD*, *psbE*, *psbF*, *psbH*, *psbI*, *psbJ*, *psbK*, *psbL*, *psbM*, *psbN*, *psbT*, *psbZ*
	NADH dehydrogenase	*ndhA*, *ndhB* *, *ndhC*, *ndhD*, *ndhE*, *ndhF*, *ndhG*, *ndhH*, *ndhI*, *ndhJ*, *ndhK*
	Cytochrome b/f complex	*petA*, *petB*, *petD*, *petG*, *petL*, *petN*
	ATP synthase	*atpA*, *atpB*, *atpE*, *atpF*, *atpH*, *atpI*
	Large subunit Rubisco	*rbcL*
Transcription and Translation	Ribosomal protein, LSU	*rpl14*, *rpl16*, rpl2 *, *rpl20*, *rpl22*, *rpl23*, *rpl32*, *rpl33*, *rpl36*
	Ribosomal protein, SSU	*rps11*, *rps12* *, *rps14*, *rps15*, *rps16*, *rps18*, *rps19*, *rps2*, *rps3*, *rps4*, *rps7* *, *rps8*
	RNA polymerase	*rpoA*, *rpoB*, *rpoC1*, *rpoC2*
	Ribosomal RNAs	*rrn16* *, *rrn23* *, *rrn4.5* *, *rrn5* *
	Transfer RNAs	*trnC-^GCA^*, *trnD-^GUC^*, *trnE-^UUC^*, *trnF-^GAA^*, *trnG-^UCC^*, *trnfM-^CAU^*, *trnH-^GUG^*, *trnI-^CAU^*, *trnK-^UUU^*, *trnL-^UAA^*, *trnL-^UAG^*, *trnA-^UGC^*, *trnP-^GGG^*, *trnP-^UGG^*, *trnQ-^UUG^*, *trnR-^UCU^*, *trnS-^GCU^*, *trnS-^GGA^*, *trnS-^UGA^*, *trnT-^GGU^*, *trnT-^UGU^*, *trnV-^UAC^*, *trnW-^CCA^*, *trnY-^GUA^*, trnI-^GAU^ *, trnL-^CAA^ *, trnM-^CAU^ *, trnN-^GUU^ *, trnR-^ACG^ *, trnV-^GAC^ *
	Maturase	*matK*
Biosynthesis	Protease	*clpP*
	Envelope membrane protein	*cemA*
	Acetyl-CoA carboxylase	*accD*
	c-type cytochrome synthesis	*ccsA*
Unknown function	Unknown group	ycf1 *, ycf2 *, *ycf3*, *ycf4*

Note: “*” indicates the duplication of genes on the plastid genome.

**Table 2:** List of genes in the *C. officinarum* chloroplast genome.

Samples	Total Length	LSC Length (GC Content)	SSC Length (GC Content)	IR Length (GC Content)	Total GC Content
HN45 (Clade I)	152,729 bp	93,687 bp (38.0%)	18,894 bp (33.9%)	40,148 bp (44.4%)	39.2%
FJ03 (Clade II)	152,620 bp	93,565 bp (37.9%)	18,863 bp (33.8%)	40,192 bp (44.4%)	39.1%
GXZG03 (Clade III)	154,066 bp	93,609 bp (37.9%)	18,873 bp (33.8%)	41,584 bp (44.3%)	39.1%
HBDY10 (Clade IV)	152,729 bp	93,687 bp (38.0%)	18,894 bp (33.9%)	40,148 bp (44.4%)	39.2%
JX28 (Clade V)	154,078 bp	93,599 bp (37.9%)	18,859 bp (33.8%)	41,620 bp (44.3%)	39.1%
GXDF2 (Clade VI)	152,729 bp	93,688 bp (38.04%)	18,893 bp (33.9%)	40,148 bp (44.4%)	39.2%
DJ249-2 (Clade VII)	153,976 bp	93,565 bp (37.9%)	18,863 bp (33.8%)	41,548 bp (44.3%)	39.2%

## Data Availability

All the data used in the present study has been included in the Supplementary Materials.

## Supplementary Materials

The following supporting information can be downloaded at: https://www.mdpi.com/article/10.3390/ijms26189229/s1.

## References

[1] Palmer J.D., Jansen R.K., Michaels H.J., Chase M.W., Manhart J.R. (1988). Chloroplast DNA variation and plant phylogeny. Ann. Mo. Bot. Gard..

[2] Daniell H., Lin C.-S., Yu M., Chang W.-J. (2016). Chloroplast genomes: Diversity, evolution, and applications in genetic engineering. Genome Biol..

[3] Palmer J.D., Stein D.B. (1986). Conservation of chloroplast genome structure among vascular plants. Curr. Genet..

[4] Shaw J., Lickey E.B., Schilling E.E., Small R.L. (2007). Comparison of whole chloroplast genome sequences to choose noncoding regions for phylogenetic studies in angiosperms: The tortoise and the hare III. Am. J. Bot..

[5] Dobrogojski J., Adamiec M., Luciński R. (2020). The chloroplast genome: A review. Acta Physiol. Plant..

[6] Lu R.-S., Li P., Qiu Y.-X. (2017). The complete chloroplast genomes of three *Cardiocrinum* (Liliaceae) species: Comparative genomic and phylogenetic analyses. Front. Plant. Sci..

[7] Zhai W., Duan X., Zhang R., Guo C., Li L., Xu G., Shan H., Kong H., Ren Y. (2019). Chloroplast genomic data provide new and robust insights into the phylogeny and evolution of the Ranunculaceae. Mol. Phylogenetics Evol..

[8] Zhang X., Rong C., Qin L., Mo C., Fan L., Yan J., Zhang M. (2018). Complete chloroplast genome sequence of *Malus hupehensis*: Genome structure, comparative analysis, and phylogenetic relationships. Molecules.

[9] Chaw S.-M., Chang C.-C., Chen H.-L., Li W.-H. (2004). Dating the monocot–dicot divergence and the origin of core eudicots using whole chloroplast genomes. J. Mol. Evol..

[10] Sun Y., Moore M.J., Zhang S., Soltis P.S., Soltis D.E., Zhao T., Meng A., Li X., Li J., Wang H. (2016). Phylogenomic and structural analyses of 18 complete plastomes across nearly all families of early-diverging eudicots, including an angiosperm-wide analysis of IR gene content evolution. Mol. Phylogenetics Evol..

[11] Moore M.J., Soltis P.S., Bell C.D., Burleigh J.G., Soltis D.E. (2010). Phylogenetic analysis of 83 plastid genes further resolves the early diversification of eudicots. Proc. Natl. Acad. Sci. USA.

[12] Wu C.-S., Wang T.-J., Wu C.-W., Wang Y.-N., Chaw S.-M. (2017). Plastome evolution in the sole hemiparasitic genus laurel dodder (*Cassytha*) and insights into the plastid phylogenomics of Lauraceae. Genome Biol..

[13] Song Y., Yu W.B., Tan Y.H., Jin J.J., Wang B., Yang J.B., Liu B., Corlett R.T. (2020). Plastid phylogenomics improve phylogenetic resolution in the Lauraceae. J. Syst. Evol..

[14] Yang Z., Ferguson D.K., Yang Y. (2023). New insights into the plastome evolution of Lauraceae using herbariomics. BMC Plant Biol..

[15] Xiao T.-W., Ge X.-J. (2022). Plastome structure, phylogenomics, and divergence times of tribe Cinnamomeae (Lauraceae). BMC Genom..

[16] Yang Z., Liu B., Yang Y., Ferguson D.K. (2022). Phylogeny and taxonomy of *Cinnamomum* (Lauraceae). Ecol. Evol..

[17] Xu J., Zhang H., Yang F., Zhu W., Li Q., Cao Z., Song Y., Xin P. (2025). Phylogeny of *Camphora* and *Cinnamomum* (Lauraceae) based on plastome and nuclear ribosomal DNA data. Int. J. Mol. Sci..

[18] Yang Z., Ferguson D.K., Yang Y. (2023). Plastome phylogeny and taxonomy of *Cinnamomum guizhouense* (Lauraceae). Forests.

[19] Li D., Lin H.-Y., Wang X., Bi B., Gao Y., Shao L., Zhang R., Liang Y., Xia Y., Zhao Y.-P. (2023). Genome and whole-genome resequencing of *Cinnamomum camphora* elucidate its dominance in subtropical urban landscapes. BMC Biol..

[20] Zhong Y., Yang A., Li Z., Zhang H., Liu L., Wu Z., Li Y., Liu T., Xu M., Yu F. (2019). Genetic diversity and population genetic structure of *Cinnamomum camphora* in South China revealed by EST-SSR markers. Forests.

[21] Gong X., Yang A., Wu Z., Chen C., Li H., Liu Q., Yu F., Zhong Y. (2021). Employing genome-wide SNP discovery to characterize the genetic diversity in *Cinnamomum camphora* using genotyping by sequencing. Forests.

[22] Meng J., Li M., Guo J., Zhao D., Tao J. (2021). Predicting suitable environments and potential occurrences for *Cinnamomum camphora* (Linn.) Presl. Forests.

[23] Zhang L., Jing Z., Li Z., Liu Y., Fang S. (2019). Predictive modeling of suitable habitats for *Cinnamomum Camphora* (L.) presl using maxent model under climate change in China. Int. J. Environ. Res. Public Health.

[24] Tian Z., Luo Q., Li Y., Zuo Z. (2020). Terpinene and β-pinene acting as signaling molecules to improve *Cinnamomum camphora* thermotolerance. Ind. Crops Prod..

[25] Chen C., Zheng Y., Zhong Y., Wu Y., Li Z., Xu L.-A., Xu M. (2018). Transcriptome analysis and identification of genes related to terpenoid biosynthesis in *Cinnamomum camphora*. BMC Genom..

[26] Chen C., Zhong Y., Yu F., Xu M. (2020). Deep sequencing identifies miRNAs and their target genes involved in the biosynthesis of terpenoids in *Cinnamomum camphora*. Ind. Crops Prod..

[27] Hou J., Zhang J., Zhang B., Jin X., Zhang H., Jin Z. (2020). Transcriptional analysis of metabolic pathways and regulatory mechanisms of essential oil biosynthesis in the leaves of *Cinnamomum camphora* (L.) Presl. Front. Genet..

[28] Ni Z., Han X., Chen C., Zhong Y., Xu M., Xu L.A., Yu F. (2021). Integrating GC-MS and ssRNA-Seq analysis to identify long non-coding RNAs related to terpenoid biosynthesis in *Cinnamomum camphora*. Ind. Crops Prod..

[29] Yang Z., Xie C., Zhan T., Li L., Liu S., Huang Y., An W., Zheng X., Huang S. (2021). Genome-wide identification and functional characterization of the trans-isopentenyl diphosphate synthases gene family in *Cinnamomum camphora*. Front. Plant Sci..

[30] Zhu C., Zhang F., Chen S., Wang K., Xiang G., Liang X., An J., Li K., Liu L. (2022). Proteomics analysis and identification of proteins related to isoprenoid biosynthesis in *Cinnamomum camphora* (L.) Presl. Forests.

[31] Kameyama Y., Furumichi J., Li J., Tseng Y.-H. (2017). Natural genetic differentiation and human-mediated gene flow: The spatiotemporal tendency observed in a long-lived *Cinnamomum camphora* (Lauraceae) tree. Tree Genet. Genomes.

[32] Chen C., Zheng Y., Liu S., Zhong Y., Wu Y., Li J., Xu L.-A., Xu M. (2017). The complete chloroplast genome of *Cinnamomum camphora* and its comparison with related Lauraceae species. PeerJ.

[33] Li P., Jia G., Xin G., Cai X. (2019). The complete chloroplast genome of *Cinnamomum camphora* (L.) Presl., a unique economic plant to China. Mitochondrial DNA Part B.

[34] Qiu M.-Y., Yang Y., Wang N., Wu X., Hu Y.-L., Zou X.-X. (2020). The re-sequencing of complete chloroplast genome of *Cinnamomum camphora* (Lauraceae) from Quanzhou, China. Mitochondrial DNA Part B.

[35] Mower J.P., Vickrey T.L. (2018). Structural diversity among plastid genomes of land plants. Adv. Bot. Res..

[36] Zhu A., Guo W., Gupta S., Fan W., Mower J.P. (2016). Evolutionary dynamics of the plastid inverted repeat: The effects of expansion, contraction, and loss on substitution rates. New Phytol..

[37] Guo Y.-Y., Yang J.-X., Bai M.-Z., Zhang G.-Q., Liu Z.-J. (2021). The chloroplast genome evolution of *Venus slipper* (Paphiopedilum): IR expansion, SSC contraction, and highly rearranged SSC regions. BMC Plant Biol..

[38] Kojoma M., Kurihara K., Yamada K., Sekita S., Satake M., Iida O. (2002). Genetic identification of cinnamon (*Cinnamomum* spp.) based on the *trnL-trnF* chloroplast DNA. Planta Medica.

[39] Chanderbali A.S., Van Der Werff H., Renner S.S. (2001). Phylogeny and historical biogeography of Lauraceae: Evidence from the chloroplast and nuclear genomes. Ann. Mo. Bot. Gard..

[40] Xia L., Wang H., Zhao X., Obel H.O., Yu X., Lou Q., Chen J., Cheng C. (2023). Chloroplast pan-genomes and comparative transcriptomics reveal genetic variation and temperature adaptation in the cucumber. Int. J. Mol. Sci..

[41] Bolger A.M., Lohse M., Usadel B. (2014). Trimmomatic: A flexible trimmer for Illumina sequence data. Bioinformatics.

[42] Jin J.-J., Yu W.-B., Yang J.-B., Song Y., DePamphilis C.W., Yi T.-S., Li D.-Z. (2020). GetOrganelle: A fast and versatile toolkit for accurate de novo assembly of organelle genomes. Genome Biol..

[43] Wick R.R., Schultz M.B., Zobel J., Holt K.E. (2015). Bandage: Interactive visualization of *de novo* genome assemblies. Bioinformatics.

[44] Krumsiek J., Arnold R., Rattei T. (2007). Gepard: A rapid and sensitive tool for creating dotplots on genome scale. Bioinformatics.

[45] Tillich M., Lehwark P., Pellizzer T., Ulbricht-Jones E.S., Fischer A., Bock R., Greiner S. (2017). GeSeq–versatile and accurate annotation of organelle genomes. Nucleic Acids Res..

[46] Kearse M., Moir R., Wilson A., Stones-Havas S., Cheung M., Sturrock S., Buxton S., Cooper A., Markowitz S., Duran C. (2012). Geneious Basic: An integrated and extendable desktop software platform for the organization and analysis of sequence data. Bioinformatics.

[47] Katoh K., Standley D.M. (2013). MAFFT multiple sequence alignment software version 7: Improvements in performance and usability. Mol. Biol. Evol..

[48] Nguyen L.-T., Schmidt H.A., Von Haeseler A., Minh B.Q. (2015). IQ-TREE: A fast and effective stochastic algorithm for estimating maximum-likelihood phylogenies. Mol. Biol. Evol..

[49] Letunic I., Bork P. (2021). Interactive Tree Of Life (iTOL) v5: An online tool for phylogenetic tree display and annotation. Nucleic Acids Res..

[50] Librado P., Rozas J. (2009). DnaSP v5: A software for comprehensive analysis of DNA polymorphism data. Bioinformatics.

[51] Frazer K.A., Pachter L., Poliakov A., Rubin E.M., Dubchak I. (2004). VISTA: Computational tools for comparative genomics. Nucleic Acids Res..

[52] Lohse M., Drechsel O., Kahlau S., Bock R. (2013). OrganellarGenomeDRAW—A suite of tools for generating physical maps of plastid and mitochondrial genomes and visualizing expression data sets. Nucleic Acids Res..

[53] Nystuen A. (2001). Lasergene 5.0.1. Biotech Softw. Internet Rep..

[54] Li H., Guo Q., Xu L., Gao H., Liu L., Zhou X. (2023). CPJSdraw: Analysis and visualization of junction sites of chloroplast genomes. PeerJ.

[55] Beier S., Thiel T., Münch T., Scholz U., Mascher M. (2017). MISA-web: A web server for microsatellite prediction. Bioinformatics.

[56] Kurtz S., Choudhuri J.V., Ohlebusch E., Schleiermacher C., Stoye J., Giegerich R. (2001). REPuter: The manifold applications of repeat analysis on a genomic scale. Nucleic Acids Res..

[57] Benson G. (1999). Tandem repeats finder: A program to analyze DNA sequences. Nucleic Acids Res..

