# Supplementary material for: Plastid Phylogenomics of Camphora officinarum Nees: Unraveling Genetic Diversity and Geographic Differentiation in East Asian Subtropical Forests
Source: Int J Mol Sci. 2025 Sep 21;26(18):9229. doi: 10.3390/ijms26189229 (PMC12471137; doi:10.3390/ijms26189229)
Supplement: Supplementary file 1 [file ijms-26-09229-s001.zip › Figure S1.pdf]

A

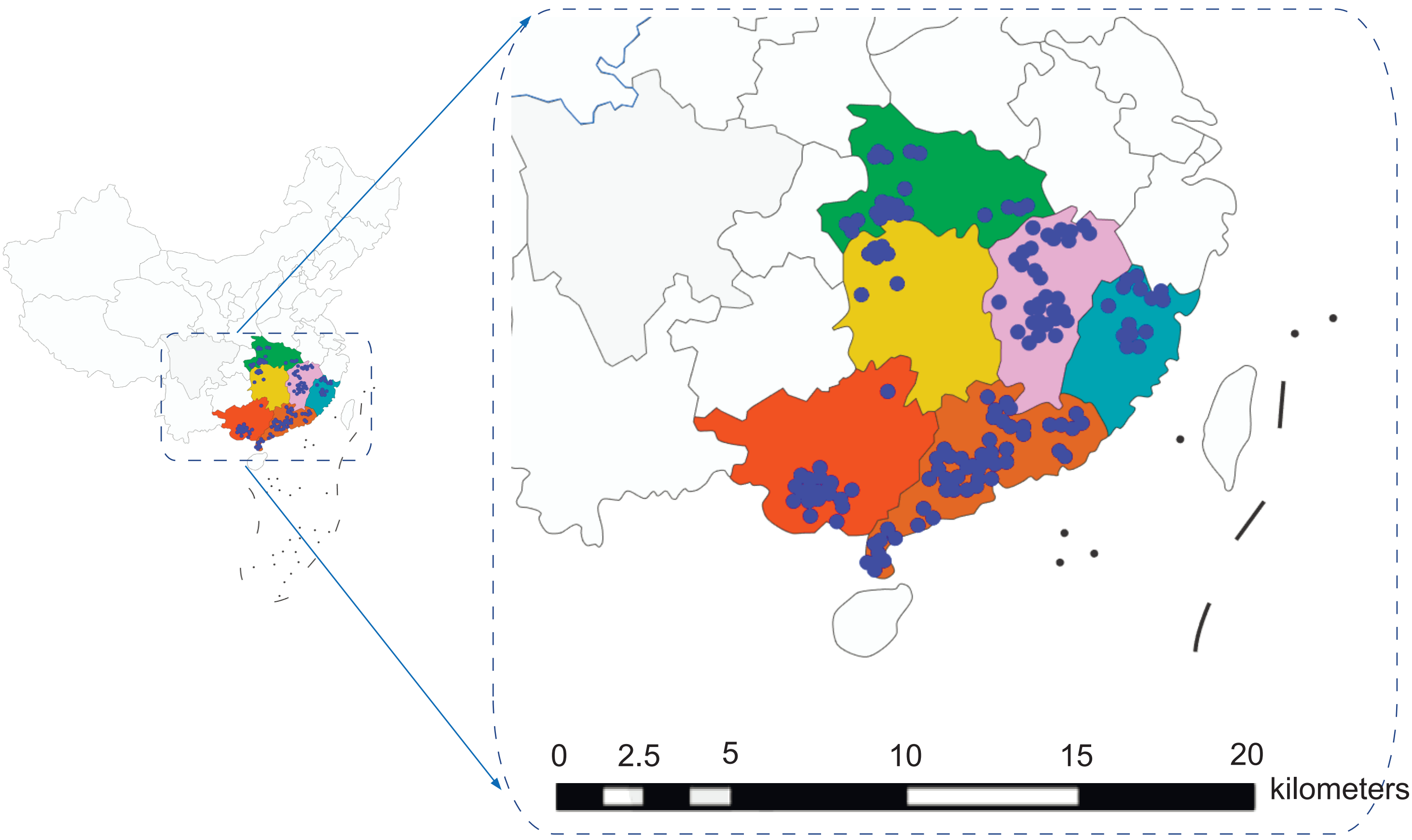

B

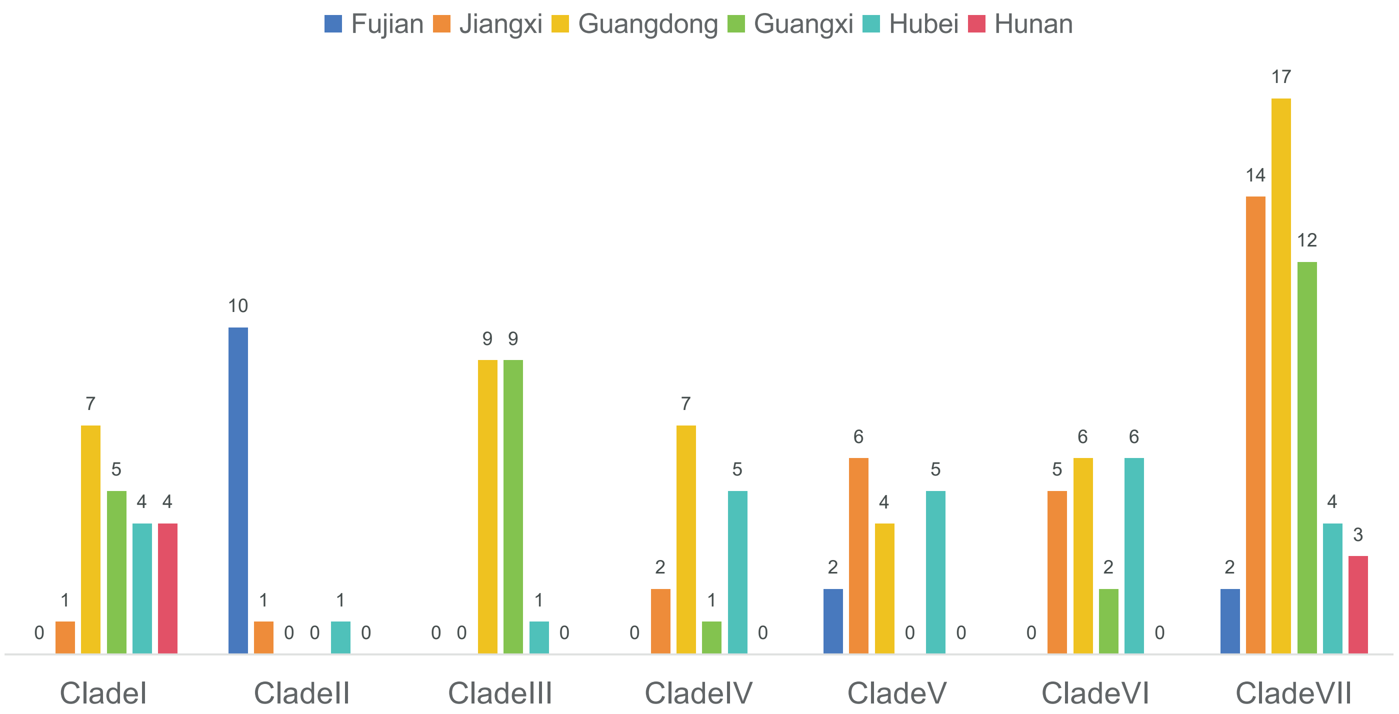

Figure S1. A. Geographic distribution of 155 *C. officinarum* samples collected in six Chinese provinces/ regions in China; B. the 155 specimens across seven phylogenetic clades in the six Chinese provinces/ regions

Supplementary S1. Sequence alignement of 155 chloroplast genome of *C. officinarum*.
